# Time of Day for Harvest Affects the Fermentation Parameters, Bacterial Community, and Metabolic Characteristics of Sorghum-Sudangrass Hybrid Silage

**DOI:** 10.1128/msphere.00168-22

**Published:** 2022-07-18

**Authors:** Zhihao Dong, Junfeng Li, Siran Wang, Dong Dong, Tao Shao

**Affiliations:** a Institute of Ensiling and Processing of Grass, College of Agro-grassland Science, Nanjing Agricultural University, Nanjing, China; University of Iowa

**Keywords:** bacterial community, fermentation parameters, metabolic characteristics, sorghum-sudangrass hybrid, time of day for harvest

## Abstract

To characterize the effects of time of day for harvest on the fermentation parameters, bacterial community, and metabolic characteristics of sorghum-sudangrass hybrid (SSG) silage, SSG (vegetative stage) harvested at 7:00 (AM), 12:00 (M), and 17:00 (PM) on three sunny days were ensiled for 1, 3, 7, 14, 30, and 60 days. Compared to AM silage, M and PM silages were characterized by delayed fermentation, unnormal lower final pH, and lower acetic acid production. In addition, PM silage contained higher residual water-soluble carbohydrates than other silages. After 60 days of ensiling, AM silage was dominated by *Lactobacillus*, whereas the bacterial communities of M and PM silages were complex and mainly composed of bacteria such as *Delftia*, *Methylobacterium-Methylorubrum*, *Enhydrobacter*, Acinetobacter, and *Bacillus*. The harvest time affected a wide range of metabolic pathways including “Metabolism” and “Cellular Processes” and “Organismal Systems” in SSG silage. Particularly, at the late stage of ensiling M silage exhibited highest relative abundances of amino acid metabolisms including “glycine, serine, and threonine metabolism,” “phenylalanine metabolism,” and lowest relative abundances of “lysine biosynthesis.” These results suggest that the time of day for harvest could affect the fermentation parameters, bacterial community, and metabolic characteristics of SSG silage. Better SSG silage characteristics could be achieved through morning harvest.

**IMPORTANCE** Ensiling is a common way for preserving green forages worldwide. Silage fermentation quality can vary greatly depending on the chemical and microbial characteristics of forage crop being ensiled. It is well documented that forages exhibit considerable variations in chemical composition and epiphytic microbiota during daylight. However, the effects of the time of day for harvest on silage fermentation is less investigated. Our results demonstrate that the time of day for harvest could affect the fermentation parameters, bacterial community, and metabolic characteristics of SSG hybrid silage. Harvesting SSG late in the day delayed fermentation process, lowered acetic acid production and final pH, and increased the residual water-soluble carbohydrates content in silage. Moreover, the delayed harvest time increased the relative abundances of bacteria such as *Delftia*, *Methylobacterium-Methylorubrum*, Acinetobacter, *Enhydrobacter*, and *Bacillus*, and amino acid metabolisms at the late stage of SSG ensiling. This study highlights the importance of diurnal changes in forage to fermentation characteristics, providing a strategy to improve silage quality through optimizing the harvest time.

## INTRODUCTION

Ensiling is a common way for preserving green forages. Yearly about 150,000 tons of silage are produced worldwide, and they are widely used as animal feeding and for biogas production. Ensiling is an anaerobic bacterial-based fermentation process, dominated by lactic acid bacteria (LAB), which produce the lactic acid required for pH decline and inhibition of non-acid-resistant undesirable microbes. The biochemistry of ensiling sounds simple, but it can be complex when interactions occur among chemical and microbial factors. Numerous factors have been reported to affect the fermentation quality of silage. Of these, material characteristics including epiphytic microbiota, dry matter (DM), water-soluble carbohydrates (WSC), and buffering capacity (BC) are most crucial (1, 2). As forage matures, chemical and microbial compositions undergo dramatic changes. Therefore, forage ensiled at different maturities differ greatly in fermentation quality (3). However, on the diurnal scale, a wide variety of plant metabolic events, such as photosynthesis and nutrient assimilation, are regulated by the circadian clock and also show the daily oscillation patterns (4). Burns et al. (5) found that the variation in chemical composition was considerable between forages cut in the morning and in the afternoon. In addition, the aerial part of plant is a highly diverse and dynamic environment (6). It is exposed to the vagaries of environmental stresses including nutrient shortage, UV radiation, and desiccation. Phyllosphere community also exhibits variations in diurnal and temporal patterns (7). Although chemical and microbial diurnal variations have long been recognized, it is surprising that few studies have investigated the effects of the time of day for harvest on the fermentation characteristics of forages.

The intensification of fodder and biofuel production systems increases the demand for forage production. Growing multipurpose crops such as sweet sorghum is gaining popularity, especially in regions that experience drought, delayed planting, and high summer temperatures, which limit corn production (8). Sorghum-sudangrass hybrid (SSG; Sorghum bicolor L.× *Sorghum sudanense* L.) is the most commonly used sorghum type because of flexible planting time, rapid growth, high yields, suitability in rotation systems, and high nutritive value (9). SSG has great carbon exchange rate over wide environmental conditions (9), and its biomass mainly accumulates in summer. During the harvest season, strong environmental fluctuations during the day may have a great impact on the chemical compositions and epiphytic microbiota of SSG. However, it is unclear how the chemical and microbial compositions change in SSG during the day and whether the diurnal changes could affect the fermentation parameters during SSG ensiling.

Silage fermentation is a dynamic process of microbial community succession and metabolite changes. Recent advances in culture-independent analyses, such as high-throughput sequencing technology, have enabled microbial communities to be defined with a degree of detail that is impossible using classical microbiology (10). Understanding of the microbial community involved in the ensiling process would provide an insight into approaches to improve the forage conservation (11, 12). In addition, more and more researches use 16S rRNA gene-based predicted functional analyses to decipher the metabolic characteristics of the bacterial community involved in ensiling (13–15). These works provided a better understanding of microbial metabolic pathways underlying the silage fermentation. Therefore, the objectives of this study were to investigate the effects of time of day for harvest on the fermentation parameters, bacterial community, and metabolic characteristics of SSG silage. The results of this study would be beneficial for precisely timing the harvest, increasing our ability to produce high-quality SSG silage.

## RESULTS AND DISCUSSION

### Forage characteristics.

Table 1 illustrates the chemical compositions and microbial counts of SSG harvested at various times within a day. Compared to AM-cut SSG, M- and PM-cut SSG had higher (*P < *0.05) DM contents, which could be linked to the loss of morning dew. SSG accumulates sucrose as the principal reserve carbohydrate (16). It was accountable for the increases in WSC contents with the delay of harvest time. Photosynthesis provides the plant energy and carbon skeletons for nitrogen assimilation. Not only photosynthate but also nitrogen component will accumulate under the light condition (17). In the present study, significant accumulation of crude protein (CP) was observed in PM-cut SSG while not in M-cut SSG, suggesting that the nitrogen assimilation rate was low between AM and M. Similarly, Burns et al. (5) observed a first decline of CP content in alfalfa from 07:00 to 13:00 h and then an increase through 19:00 h. The neutral detergent fiber (NDF) and acid detergent fiber (ADF) contents decreased in PM-cut SSG than in AM- and M-cut SSG, which could be associated with the dilutive effect of carbohydrate accumulation on other components. This was consistent with the results of Burns et al. (5) and Guo et al. (18).

**TABLE 1:** Chemical compositions and microbial counts of SSG harvested at various times within a day^*a*^

Item	07:00 h (AM)	12:00 h (M)	17:00 h (PM)	SEM	*P* value
Chemical composition (g/kg DM, unless stated otherwise)					
Dry matter (% FW)	19.6*^b^*	20.8*^a^*	21.8*^a^*	0.471	0.009
Crude protein	58.5*^b^*	51.3*^b^*	69.3*^a^*	3.640	0.008
Water-soluble carbohydrates	78.0*^c^*	106*^b^*	139*^a^*	12.17	0.013
Neutral detergent fiber	642*^a^*	636*^a^*	560*^b^*	12.28	0.001
Acid detergent fiber	410*^a^*	411*^a^*	346*^b^*	9.561	0.001
Buffering capacity (mEq/kg DM)	133	109	119	2.990	0.210
Microbial counts (log_10_CFU/g FW)					
Lactic acid bacteria	6.67*^a^*	2.52*^b^*	<2.00*^c^*	0.749	0.001
Aerobic bacteria	7.67*^a^*	8.08*^a^*	6.97*^b^*	0.163	0.010
Yeast	7.21*^a^*	6.70*^a^*	5.77*^b^*	0.179	<0.001
Coliform	7.18*^a^*	7.60*^a^*	3.80*^b^*	0.562	0.003

a DM, dry matter; FW, fresh weight; CFU, colony forming unit; mEq, milligram equivalent; SEM, standard error of means; Means with different lowercase superscript letters (a to c) are significantly different among harvest times (*P *< 0.05).

Phyllosphere microbes exist mainly on leaf surfaces, a hostile location for microbial colonization. The population and community of phyllosphere bacteria are affected by the fluctuations in the nutritional conditions of the phyllosphere, which is characterized by rapid changes in temperature, relative humidity of the environment, and UV dose (19, 20). As shown in Table 1, lactic acid bacteria (LAB) counts decreased more than other microbes with the delay of harvest time, reflecting their weakest ability against the diurnal fluctuations. LAB have complex nutritional requirements and lack the strategy to cope with the adverse environmental factors (21, 22). It was assumed that UV radiation might be the most important environmental factor in affecting LAB’s population because they are usually observed to be in lower counts in the top of the forage plant (23).

### Fermentation parameters, chemical composition, and microbial counts.

The fermentation parameters during ensiling of SSG harvested at various times within a day are shown in Table 2. The initial LAB count of preensiled forage is a critical factor in producing high-quality silage. Low LAB initial counts imply more duplication cycles required to reach the threshold number for initiating intense lactic fermentation. This explained why higher (*P < *0.05) pH and lower (*P < *0.05) lactic acid contents were observed in M and PM silages compared to AM silage during the initial 7 days of ensiling. Although there were lags in fermentation, silages harvested at various times showed no differences in pH and lactic acid contents after 7 days. It was because the high WSC content in SSG provided sufficient fermentable sugars for LAB and sustained the desirable fermentation type. Lactic acid is the main driver of lowering pH in silage. Generally speaking, desired pH values is approximately 3.8 to 4.2 for high-quality silage. However, all SSG silages had extremely low pH values less than 3.50 after 60 days of ensiling. Moreover, compared to AM silage, the final pH values of M and PM silages were even lower (3.18 for M and 3.16 for PM), although there was no difference in lactic acid production. This unnormal pH values was likely associated with the presence of strong acids in silage. Sorghum are the forage type conductive to accumulate high levels of nitrate (24). During ensiling microorganisms such as enterobacteria can convert nitrate to nitrogen dioxide, which reacts with water to form nitric acid. In this study, although nitrogen dioxide was not measured, the lower pH values in M and PM silages might be an indication of greater breakdown of nitrate to nitrogen dioxide. This could be a potential danger to human and livestock because nitrogen dioxide produced during ensiling is a “toxic silo gas,” which would damage the lung and cause pulmonary edema, bronchiolitis, and even death in severe cases (25).

**TABLE 2:** The fermentation parameters during ensiling of SSG harvested at various times within a day^*a*^

Item/harvest time	Ensiling day (d)						SEM	*P* value		
	1	3	7	14	30	60		H	D	H × D
pH value										
7:00 (AM)	4.15*^b^*^,^*^A^*	3.65*^c^*^,^*^B^*	3.53*^B^*^,^*^C^*	3.64^*B*^	3.65^*B*^	3.43^*a*,*C*^	0.081	0.003	<0.001	<0.001
12:00 (M)	5.03*^a^*^,^*^A^*	3.86*^b^*^,^*^B^*	3.49^*B*,*C*^	3.58^*B*^	3.54^*B*,*C*^	3.18^*b*,*C*^				
17:00 (PM)	5.16*^a^*^,^*^A^*	4.16*^a^*^,^*^B^*	3.53^*C*^	3.59^*C*^	3.49^*C*^	3.16^*b*,*D*^				
Lactic acid (g/kg DM)										
7:00 (AM)	41.1*^a^*^,^*^B^*	71.7^*a*,*A*^	82.4*^A^*	75.2*^A^*	68.5*^A^*	80.1*^A^*	8.046	0.550	<0.001	0.25
12:00 (M)	19.4*^b^*^,^*^C^*	44.1^*b*,*B*,*C*^	82.6*^A^*	91.2*^A^*	72.1*^A^*^,*B*^	80.1*^A^*				
17:00 (PM)	19.6*^b^*^,^*^B^*	37.7^*b*,*B*^	75.6*^A^*	85.2^*A*^	64.0^*A*^	67.3^*A*^				
Acetic acid (g/kg DM)										
7:00 (AM)	15.0*^a^*^,^*^B^*	18.0^*a*,*B*^	20.5^*a*,*B*^	19.7^*a*,*B*^	32.3^*a*,*A*^	22.6^*a*,*B*^	1.485	<0.001	<0.001	0.001
12:00 (M)	10.5^*b*^^,*C*^	12.3*^b,B,C^*	13.5^*b*,*A*,*B*^	15.4^*b*,*A*^	15.2^*b*,*A*^	13.7^*b*,*A*,*B*^				
17:00 (PM)	9.85*^b^*^,^*^B^*	10.0^*b*,*B*^	14.5^*b*,*A*^	14.6^*b*,*A*^	13.7^*b*,*A*^	12.3^*b*,*A*,*B*^				
Lactic acid/acetic acid										
7:00 (AM)	2.74*^a^*^,^*^A^*^,^*^B^*	4.00^*A*^	4.01^*b*,*A*^	3.84^*b*,*A*,*B*^	2.44^*b*,*B*^	3.54^*b*,*A*,*B*^	0.538	<0.001	<0.001	0.037
12:00 (M)	1.82*^b,C^*	3.65^*B,C*^	6.05^*a*,*A*^	5.98^*a*,*A*^	4.85^*a*,*A*,*B*^	5.80^*a*,*A*^				
17:00 (PM)	2.00^*a,b,C*^	3.79^*B*^	5.25^*a*,*b*,*A*,*B*^	5.84^*a*,*A*^	4.66^*a*,*A*,*B*^	5.44^*a*,*A*^				
Propionic acid (g/kg DM)										
7:00 (AM)	11.1*^a^*^,^*^A^*	10.7^*a*,*A*,*B*^	10.6^*A*,*B*^	10.2^*a*,*B*^	11.2^*a*,*A*^	10.5^*a*,*A*,*B*^	0.284	<0.001	0.001	0.183
12:00 (M)	9.40*^b^*^,^*^B^*	9.29^*b*,*B*^	9.93^*B*^	9.72^*a*,*b*,*B*^	10.9^*a*,*b*,*A*^	9.86^*a*,*B*^				
17:00 (PM)	9.54*^b^*^,^*^A^*^,^*^B^*	8.89^*b*,*A*,*B*^	9.61^*A*,*B*^	9.38^*b*,*A*,*B*^	9.88^*b*,*A*^	8.68^*b*,*B*^				
Ethanol (g/kg DM)										
7:00 (AM)	14.8*^a^*	21.6	15.8	15.3^*a*^	15.4^*b*^	19.6	4.377	0.037	0.249	0.507
12:00 (M)	12.4*^a^*^,^*^b^*	32.1	14.5	17.1^*a*^	22.8^*a*^	16.9				
17:00 (PM)	11.4*^b^*	10.2	13.4	11.6^*b*^	14.4^*b*^	14.8				

a WSC, water-soluble carbohydrates; DM, dry matter; H, harvest time; D, ensiling day; H × D, the interaction between harvest time and ensiling day; SEM, standard error of means; Means with different lowercase superscript letters (a to c) differ significantly among harvest times (*P *< 0.05); Means with different uppercase superscript letters (A to D) differ significantly among ensiling days (*P *< 0.05).

Acetic acid is the acid found in the second highest content in silage, generally originating from the metabolism of hetero-fermentative LAB in well-preserved silages (2). The acetic acid contents increased in AM silage, indicating the activity of heterofermentative LAB during ensiling. The fact that the first increase and then decrease in lactic/acetic acid ratios also confirmed that there was a remarkable conversion from homolactic fermentation to heterolactic fermentation in AM silage during ensiling. Heterolactic fermentation is less efficient than homolactic fermentation and may cause some DM loss. However, acetic acid produced in this process is more antimycotic than lactic acid (26). For silages of corn and sorghum that are very sensitive to aerobic deterioration, the increases in acetic acid contents are valuable because small increases in fermentative DM losses can be readily offset by substantial improvement in aerobic stability of silage during feed-out (26). By contrast, M and PM silages maintained low acetic acid contents and high lactic/acetic acid ratios, suggesting the predominance of homolactic fermentation during ensiling.

The propionic acid and butyric acid are unacceptable fermentation products in silages given that their generation is an energy-waste metabolism. The absence of butyric acid and little production of propionic acid suggested that extensive secondary fermentation did not occur during ensiling. Ethanol is also undesirable in preserving forage because its production causes extremely high losses of DM and energy. Over 30 to 40 g/kg DM production is usually considered to be associated with the action of yeasts (1). In the present study, all silages had an ethanol content less than 20 g/kg DM, suggesting that microbes such as heterolactic acid bacteria and enterobacteria are the main bacteria responsible for the ethanol production in SSG silage.

The chemical compositions and microbial counts during ensiling of SSG harvested at various times within a day are given in Table 3. The DM contents were statistically and numerically higher in M and PM silages than AM silage, which was consistent with DM contents in the raw materials. During ensiling, proteolysis by plant and microbial enzymes will lower the nutritive value of ensiled forage by degrading protein into nonprotein fractions, such as peptides, free amino acid, and NH_3_. Typically, satisfying level of NH_3_-N in quality silage should be less than 100 to 150 g/kg total nitrogen (TN) (1). The low NH_3_-N contents of all SSG silages suggested that forage protein was well preserved during ensiling. The WSC decreased rapidly during the initial 7 days, which coincided with the lactic acid production and pH declines. It suggested that WSC was mainly consumed by LAB for lactic acid production. After 60 days of ensiling more residual WSC was observed in the PM silage (*P < *0.05). It could be due to the excessively high WSC content in PM-cut SSG, resulting in more WSC retained in the final silage. Higher residual WSC is nutritionally desirable as it is rapidly digestible in the rumen (27). However, it also carries a higher risk of yeast spoilage at silage opening considering that not enough antifungal agent acetic acid was present in the PM silage.

**TABLE 3:** The chemical compositions and microbial counts during ensiling of SSG harvested at various times within a day^*a*^

Item/harvest time	Ensiling day (d)						SEM	*P* value		
	1	3	7	14	30	60		H	D	H × D
DM (% FW)										
7:00 (AM)	18.7^*b*,*A*,*B*^	19.0^*c,A,B*^	19.0^*b*,*A*,*B*^	19.7^*b*,*A*^	18.1^*b*,*B*^	19.3^*b*,*A*,*B*^	0.520	<0.001	<0.001	0.370
12:00 (M)	21.5^*a*,*A*^	21.6^*b*,*A*^	20.3^*a*,*b*,*A*,*B*^	20.8^*a*,*b*,*A*,*B*^	19.4^*a*,*b*,*B*^	21.1^*a*,*b*,*A*,*B*^				
17:00 (PM)	21.3^*a*,*B*^	22.8^*a*,*A*,*B*^	21.2^*a*,*B*^	22.0^*a*,*A*,*B*^	20.8^*a*,*B*^	23.4^*a*,*A*^				
NH_3_-N (g/kg TN)										
7:00 (AM)	58.4	90.3	76.0	56.8	68.1	88.2	2.481	0.134	0.016	0.169
12:00 (M)	48.2	54.5	69.1	83.9	64.5	75.5				
17:00 (PM)	39.0^*C*^	64.4^*B*^	90.1^*A*^	61.9^*B*,*C*^	45.8^*B*,*C*^	61.2^*B*,*C*^				
WSC (g/kg DM)										
7:00 (AM)	45.2^*c,A*^	16.5^*c,B,C*^	8.29^*c,C*^	11.1^*b*,*B*,*C*^	11.2^*b*,*B*,*C*^	20.6^*b*,*B*^	8.492	<0.001	<0.001	0.012
12:00 (M)	81.9^*b*,*A*^	32.0^*b*,*B*^	22.4^*b*,*B*^	20.2^*b*,*B*^	21.9^*b*,*B*^	26.2^*b*,*B*^				
17:00 (PM)	138^*a*,*A*^	87.1^*a*,*B*,*C*^	49.5^*a,C*^	76.1^*a*,*B*,*C*^	80.0^*a*,*A*,*B*^	55.9^*a*,*C*^				
LAB, (log_10_ cfu/g FW)										
7:00 (AM)	7.82^*a*,*A*^	8.96^*A*^	8.42^*A*^	8.34^*A*^	4.72^*a*,*B*^	5.32^*a*,*B*^	<0.001	<0.001	<0.001	<0.001
12:00 (M)	6.04^*b*,*C*^	8.97^*A*^	8.71^*A*^	8.26^*B*^	<2.00^*b*,*D*^	<2.00^*b*,*D*^				
17:00 (PM)	6.17^*b,C*^	8.64^*A*^	8.73^*A*^	8.13^*B*^	<2.00^*b*,*D*^	<2.00^*b*,*D*^				
Coliform (log_10_ cfu/g FW)										
7:00 (AM)	<2.00^*b*,*B*^	<2.00^*b*,*B*^	<2.00^*b*,*B*^	2.97^*A*^	<2.00^*B*^	<2.00^*B*^	0.764	<0.001	<0.001	<0.001
12:00 (M)	6.62^*a*,*A*^	4.44^*a*,*A*,*B*^	4.58^*a*,*A*,*B*^	2.47^*B*^	<2.00^*C*^	<2.00^*C*^				
17:00 (PM)	6.73^*a*,*A*^	3.61^*a*,*B*^	<2.00^*b*,*C*^	3.57^*B*^	<2.00^*C*^	<2.00^*C*^				
Aerobic bacteria (log_10_ cfu/g FW)										
7:00 (AM)	<2.00^*c*,*C*^	<2.00^*C*^	3.18^*A*^	2.99^*a*,*B*^	<2.00^*B*,*C*^	<2.00^*B*,*C*^	0.912	0.002	<0.001	<0.001
12:00 (M)	7.51^*a*,*A*^	<2.00^*C*^	3.71^*A*^	2.97^*a*,*B*^	3.64^*B*^	<2.00^*C*^				
17:00 (PM)	7.25^*b*,*A*^	2.03^*B*^	5.36^*A*^	2.40^*b*,*B*^	<2.00^*C*^	<2.00^*C*^				

a DM, dry matter; LAB, lactic acid bacteria; TN, total nitrogen; CFU, colony forming unit; FW, fresh weight; H, harvest time; D, ensiling day; H × D, the interaction between harvest time and ensiling day; SEM, standard error of means; Means with different lowercase superscript letters (a to c)differ significantly among harvest times (*P *< 0.05); Means with different uppercase superscript letters (A to D) differ significantly among ensiling days (*P *< 0.05).

The LAB is a group of Gram-positive, low guanine-cytosine containing, nonmotile, non-spore- forming, aerotolerant bacteria, which produce lactic acid as the major end product of the carbohydrate’s fermentation. The rapid increase in LAB counts during the initial stages of ensiling indicated that LAB effectively utilized WSC for their proliferation (Table 3). However, LAB counts decreased dramatically after 30 days of ensiling, suggesting that the extremely low pH developed in the silage suppressed not only undesirable bacteria but also LAB. Similarly, Sun et al. (28) have reported a decrease in LAB counts in whole-plant corn silage when pH declined to 4.0 at the late stage of ensiling. The lower pH values might also explain the lower LAB counts in M and PM silages than AM silage after 30 days of ensiling. The acidic and anaerobic conditions of silage are unsuitable for most undesirable bacteria. It was thus expected for the overall decreasing tendency in counts of coliform and aerobic bacteria during ensiling. The fast-initial acidification is the key to inhibiting the growth of coliform in silage (24). The slower rates of pH decline may result in higher coliform counts in M and PM silages compared to AM silage at the initial stages of ensiling.

### Bacterial diversity and community.

**FIG 1:**
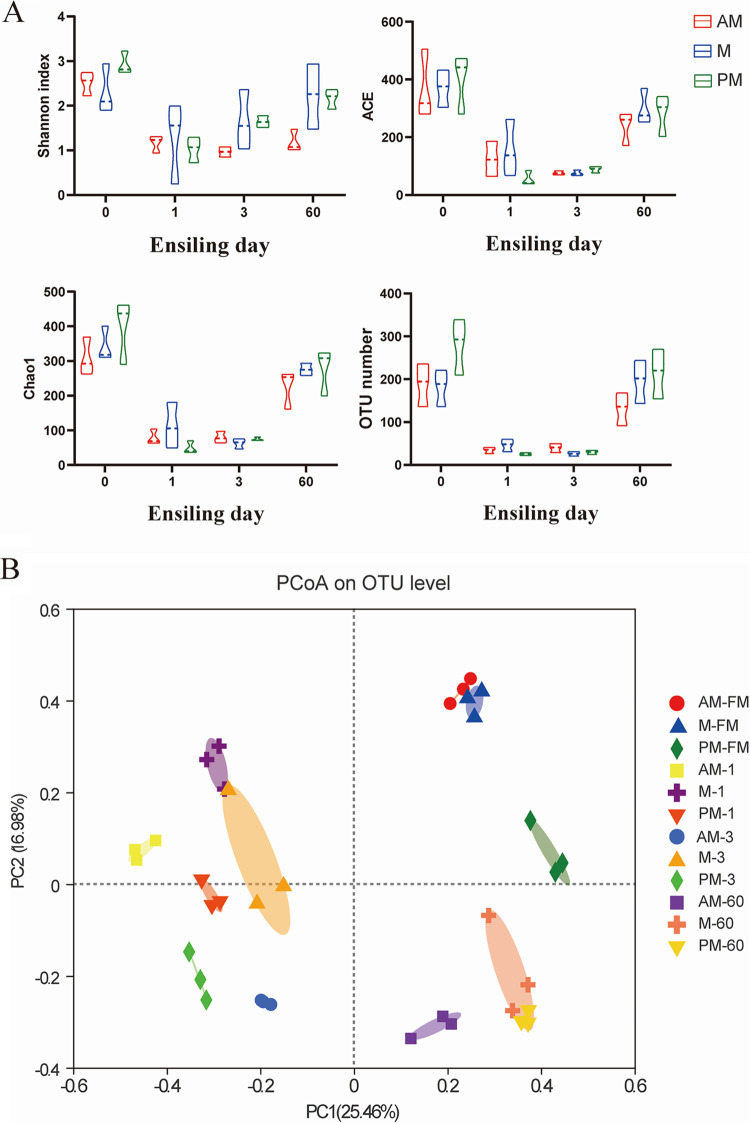
Bacterial diversity during SSG ensiling. AM, 7:00; M, 12:00; PM, 17:00. FM. fresh material. Arabic numbers indicate the days of ensiling. (A) Alpha diversity estimators for SSG silage harvested at various times within a day. (B) Principal coordinates analysis (PCoA) of bacterial communities on OTU level.

Ensiling is a bacterial-driven process in which the types and abundances of bacteria involved play a critical role in fermentation quality. In this study, fresh-cut SSG and silage samples on days 1, 3, and 60 of ensiling were selected for bacterial community analysis considering that these time points may better reflect different fermentation stages. As a result, a total of 1,870,682 high-quality reads from the V3 to V4 hypervariable region of bacterial 16S rRNA gene sequence were obtained. The average length of the reads was 426 bp. Based on a 97% sequence identity threshold, these reads were clustered into 1,210 operational taxonomic units (OTUs). Generally, the diversely microbial communities in crops were formed in the field, when the stable environment is disturbed, better-adapted microbes would dominate in the new environment. Analogously, silage is new environment for various microorganisms present in fresh crops. It favors the growth of LAB while excludes some microbiota that are unsuitable for the fermentation system. This explained the initial drops in diversity index (Shannon) and species richness estimates (ACE, Chao1, and OTU number) after the onset of ensiling (Fig. 1A). After 60 days of ensiling, bacterial diversity index was higher in M and PM silages than AM silage. High-quality silage is generally characterized by low bacterial diversity due to LAB dominance. The higher bacterial diversity indicated complex bacterial communities and was likely associated with the declines of LAB population at the late stage of ensiling.

Principal coordinate analysis (PCoA) analysis was used to explore the similarities or dissimilarities of bacterial communities (Fig. 1B). The AM- and M-cut fresh SSG plots were clustered together, suggesting a high degree of similarity of bacterial communities. The AM- and M-cut SSG cluster were far from that of PM-cut SSG, suggesting that bacterial communities of PM-cut SSG differed from those of AM- and M-cut SSG. Clear separations between fresh and silage samples suggested that ensiling greatly altered the bacterial community. The AM, M, and PM silage samples distinctly clustered away from each other during the first 3 days, suggesting that harvest time had a clear impact on the bacterial community during the early stages of ensiling. After 60 days of ensiling, M and PM silage samples clustered together and were far from AM silage samples. It suggested that M and PM silages had similar final bacterial communities, which were different from that of AM silage. Silage bacteria are derived and developed from the epiphytic microbiota. Therefore, silage bacteria are highly relevant with the bacterial species in epiphytic microbiota. It has to be noted that, although AM- and M-cut SSG had a similar epiphytic microbiota, they were also different in bacterial community composition during ensiling. It suggested that the bacterial community structure during ensiling was largely shaped by the population dynamics of a small group of bacteria (e.g., LAB) rather than the predominant bacteria in the epiphytic microbiota.

**FIG 2:**
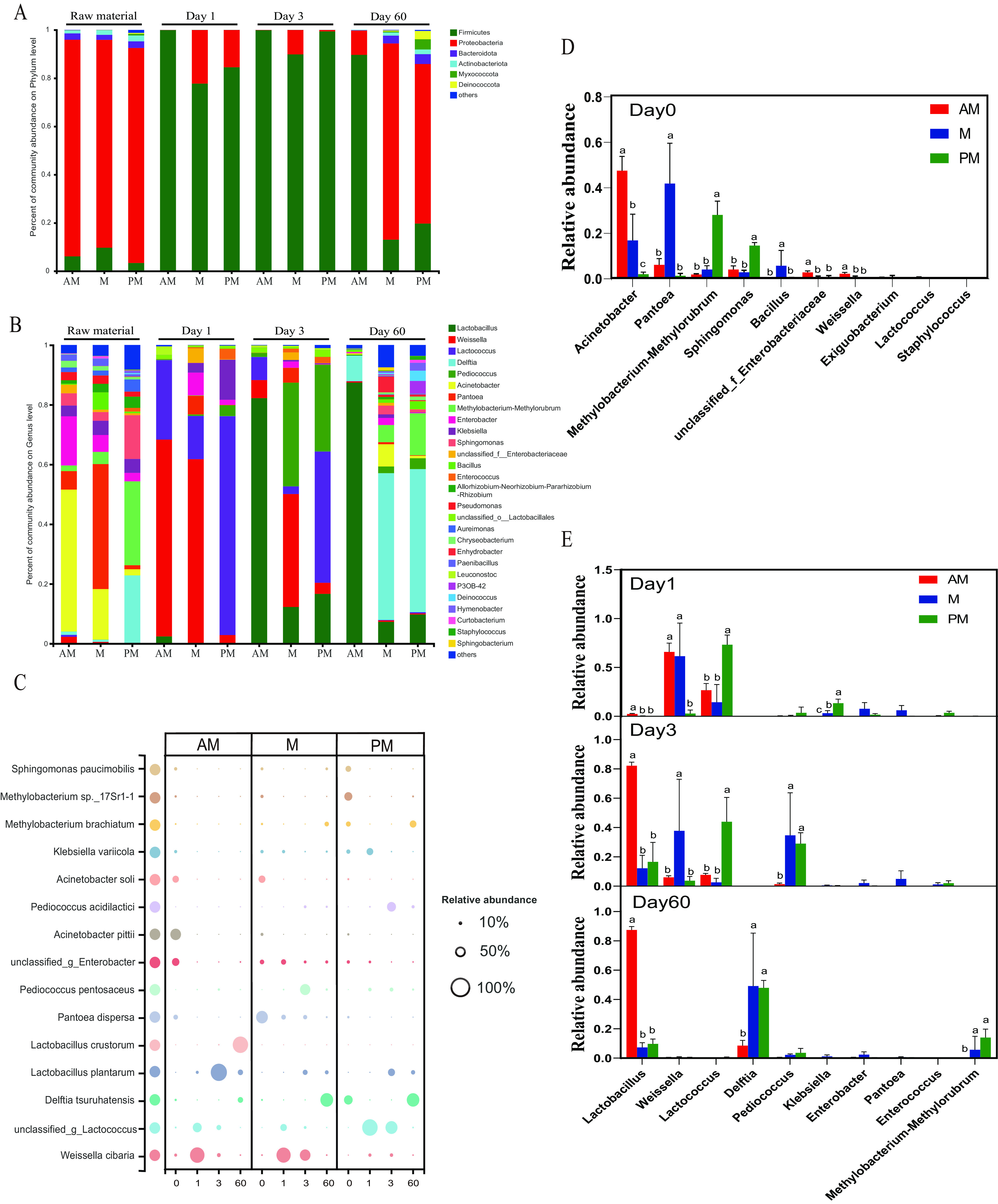
The structures and comparison of bacterial community of SSG silage harvested at various times. The bacterial community structures on phylum (A), genus (B), and species (C) level in fresh and ensiled SSG. Phyla and genera detected at less than 1.0% of total sequence reads are not included. Only species ranked in top 15 were presented. AM, 7:00; M, 12:00; PM, 17:00. Statistical comparison of the relative abundance of bacterial communities (top 10) on genus level in raw material (D) and silage (E). Error bars denote the standard error of means. Different lowercase letters (a–c) above each bar denote significant differences among harvest times according to Tukey’s test (*P < *0.05).

*Proteobacteria* is a major phylum of Gram-negative bacteria that includes a wide variety of pathogenic genera. As displayed in Fig. 2A, all fresh SSG were dominated by *Proteobacteria*, suggesting the presence of large number of undesirable microorganisms in SSG. After ensiling, *Firmicutes* increased dramatically and became the most abundant phylum in SSG silage. Microorganisms belonging to *Firmicutes* are crucial acid-hydrolytic microbes under anaerobic circumstances. They can produce various proteases, lipases, cellulases and other enzymes. As all LAB belong to *Firmicutes*, the dramatic increase in the abundance of *Firmicutes* indicated rapid LAB development during ensiling. However, for M and PM silages, *Proteobacteria* again replaced *Firmicutes* as the dominant phylum at the end of ensiling, suggesting the failure of LAB to dominate the microbiota in the final silage.

The bacterial community structures on genus level in raw material and silage are shown in Fig. 2B. The most dominant genus in AM-cut SSG was Acinetobacter, while in M and PM-cut SSG were *Pantoea* and *Methylobacterium*-*Methylorubrum*, respectively. It suggested that epiphytic microbiota structure was highly dynamic during the day. The relative abundances of Acinetobacter, *unclassified_f_Enterobacteriaceae*, and *Weissella* decreased with the delay of harvest time (Fig. 2D), which might be a result of extensive killing of these bacteria under diurnal environmental stresses. By contrast, the relative abundances of bacteria, such as *Methylobacterium-Methylorubrum*, *Pantoea*, *Bacillus*, and *Sphingomonas*, increased during the day (Fig. 2D). *Methylobacterium-Methylorubrum*, *Pantoea*, and *Bacillus* are composed of a variety of pigmented bacteria and most species are shown to be UV resistant (29, 30). *Sphingomonas* can produce TonB-dependent transport systems to scavenge various nutrients which are present at low concentrations (31). The enrichment of these bacteria could be attributed to their efficient strategies to resist the diurnal environmental stresses. *Pantoea* and *Bacillus* were most abundant in M-cut SSG, while *Methylobacterium-Methylorubrum* and *Sphingomonas* were most abundant in PM-cut SSG. This suggested that the main environmental driver of population dynamics may vary between these two bacterial groups.

Establishment of a microbial community dominated by LAB during the fermentation phase is the key to successful ensiling. In agreement with other forage crops (32), LAB represent only a very small fraction of the epiphytic microbiota of SSG (<3%) (Fig. 2B). After the onset of ensiling, *Weissella* and *Lactococcus* quickly dominated the microbiota. On day 1 of ensiling, *Weissella* was more abundant in AM and M silage, and *Lactococcus* was more abundant in PM silage (Fig. 2E). *Weissella* is strictly a heterofermentative LAB, producing a mixture of lactic acid and acetic acid by metabolizing WSC, and it is the prevailing identifiable genus in untreated silages (33). *Lactococcus* was mostly identified as homofermentative LAB and was abundant in a wide variety of silages (34). *Weissella* and *Lactococcus* are LAB contributing to the initial decline in silage pH, but they are not as acid resistant as *Lactobacillus*. After 3 days of ensiling, *Lactobacillus* started to dominate the AM silage. Members of *Lactobacillus* are Gram-positive, non-spore-forming, rods, or coccobacilli. It can quickly ferment a wide variety of substrates, contributing substantially to the acid accumulation and pH decline to levels <4.5 (35). Therefore, *Lactobacillus* is the main active ingredient of silage inoculant often used to promote successful ensiling (36, 37). Compared to AM silage, M and PM silages had higher abundances of *Pediococcus* on d 3 of ensiling. *Pediococcus* is homofermentative LAB and grow faster than *Lactobacillus* (38). Some species of *Pediococcus* show high acid tolerance (39) and are also effective in improving silage quality whereby lactic acid production (40). At the late stage of ensiling, the dominating role of Lactobacillus plantarum was replaced by Lactobacillus crustorum in AM silage (Fig. 2C). Lactobacillus plantarum is practically classified as homofermentative LAB fermenting hexoses to exclusively lactic acid. Nevertheless, Lactobacillus crustorum is heterofermenters producing mixed acids including lactic acid and acetic acid (41). The prevalence of Lactobacillus crustorum in AM silage could explain why metabolic pathway shifted from homofermentative to heterofermentative at the late stage of ensiling.

The relative abundance of Klebsiella increased from AM to PM silage on day 1 (Fig. 2E). Klebsiella is a group of rod-shaped bacteria of family enterobacteriaceae. Its higher abundance was consistent with the higher enterobacterial counts at the initial stage of ensiling in M and PM silages. Enterobacteria represent a minor part of the epiphytic microbiota, but they usually present in significantly higher numbers than LAB in the initial few hours after the onset of ensiling (28). Fast initial acidification is the key to controlling their growth in silage, and when fermentation is delayed, they may develop into large numbers (24). Some species of Klebsiella are found to produce 1,3-propanediol during fermentation (42), and some produce propanediol and butyric acid, which are not desirable indicators for silage quality (43). Klebsiella also destabilizes the aerobic stability of silage, and some species are opportunistic pathogens, which can cause mastitis in animals (44). *Delftia* dominated the microbiota of M and PM silages at the end of ensiling (Fig. 2B and E). *Delftia* was rarely reported in silages, and this is the first report on its dominance in silage. Members of *Delftia* are nonfermentative, chemo-organotrophic rods that are commonly found in soil, in water, and on plants. It can degrade phenolic compounds and aniline in polluted soil and water (30, 45). Also, *Delftia* has been reported as a nitrate reducer contributing to the reduction of nitrate to nitrite in wastewater and activated sludge (46). However, unlike enterobacteria, *Delftia* cannot further reduce nitrite. This may directly result in considerable accumulation of nitrite in the silage. Under acidic conditions, nitrite is chemically unstable and can be converted to nitrogen oxides, such as nitrogen dioxide, nitrous oxide and nitric oxide (1), which may account for the unnormal lower pH values in M and PM silages at the late stage of ensiling.

*Methylobacterium-Methylorubrum* was more abundant in M and PM silages than AM silage at the end of ensiling (Fig. 2E). It was consistent with the higher abundances in the M and PM-cut SSG, suggesting that some diurnally enriched bacteria could survive in silage and possibly serve as a source of bacteria for silage at the late stage of ensiling. *Methylobacterium-Methylorubrum* is Gram-negative, rod-shaped, and strictly aerobic bacteria that can utilize methanol and other reduced one-carbon compounds via the serine pathway. Usually, microbes of this genus are neutrophilic and their abundance decreases as pH declines. It is unclear why *Methylobacterium-Methylorubrum* was still detected in large quantities in M and PM silages (5.74% in M and 14.1% in PM) at the end of ensiling. Similarly, *Methylobacterium-Methylorubrum* has been also found to be abundant in alfalfa silage after 100 days of ensiling (47). However, to date its role in silage fermentation is less known.

Despite not being statistically significant, other bacteria, such as Acinetobacter, *Enhydrobacter*, and *Bacillus*, were more abundant in M and PM silages than AM silage (Fig. 2B). Acinetobacter species are aerobic, nonfermenting bacteria that can be found in different environments. Some Acinetobacter species can survive in anaerobic environment in the presence of acetate as a substrate (48). They were reported to link the aerobic deterioration (49) and DM losses (50) in silages. *Enhydrobacter* is a Gram-negative, nonmotile, facultative anaerobe belonging to the Moraxellaceae family. This genus differs from other species in intracellular gas vacuoles (51). *Enhydrobacter* ferments sugars anaerobically and organic acids aerobically, suggesting that the presence of this bacterium may be undesirable for silage quality and aerobic stability at silage opening. Members of *Bacillus* are endospore-forming aerobic or facultatively anaerobic bacteria. Some species of this genus can produce cellulase or other enzymes like α-amylase and feruloyl esterase to degrade plant structural carbohydrates during ensiling (52, 53). The *Bacillus* species also produces lactic acid but is generally less efficient than LAB. The genus *Bacillus* is a diverse group of spore-forming bacteria. The spores can contaminate milk after passing through the alimentary tract of dairy cows (54).

### Interaction between bacteria and relationship between bacterial community and fermentation parameters.

**FIG 3:**
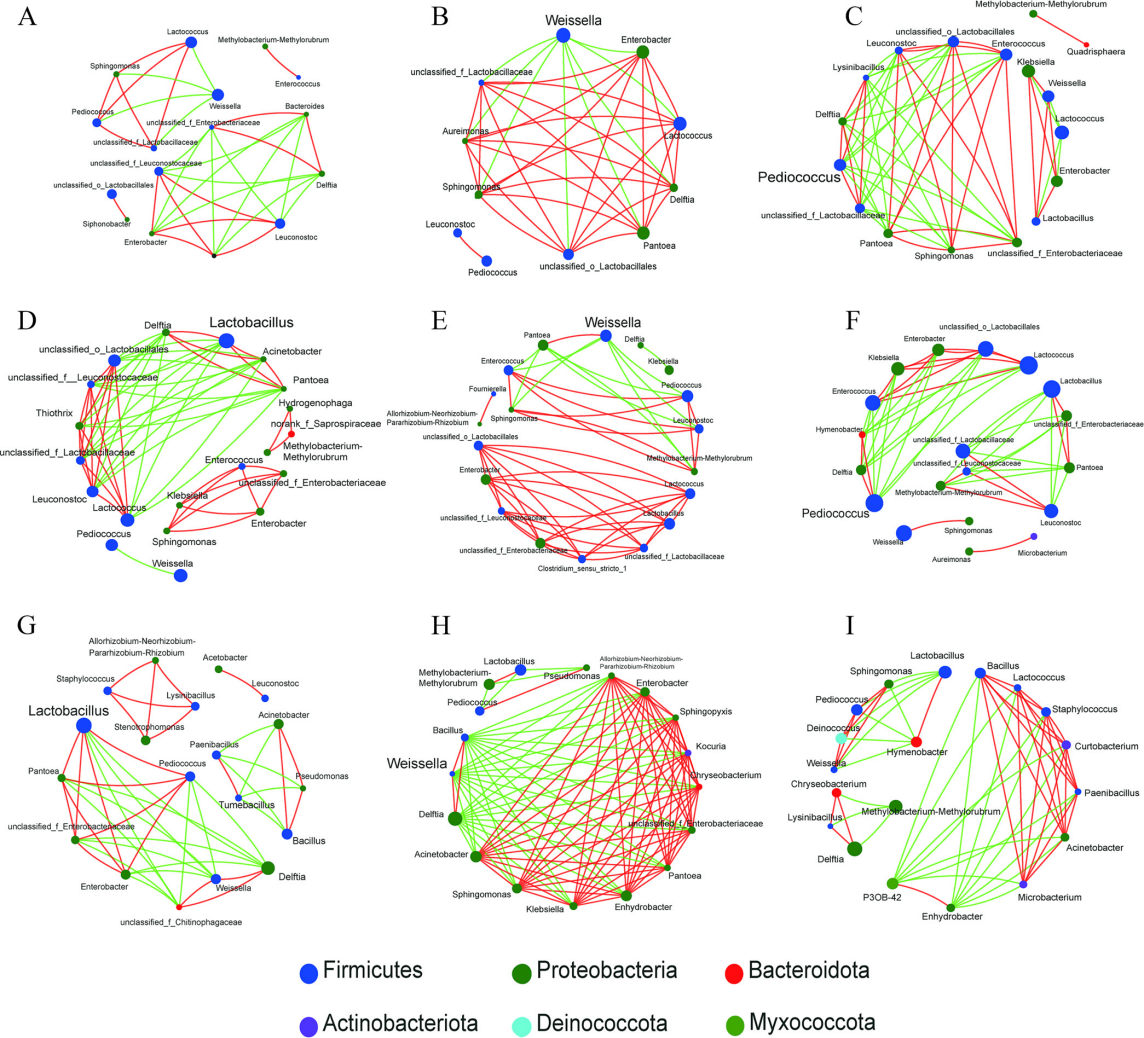
Interaction networks of top 20 genera during SSG ensiling. A connection stands for a significant (*P < *0.05) and strong (Spearman’s |*P*| >0.6) correlation. Size of each node is proportional to the relative abundance, and the nodes are colored by phylum. The color of the edges corresponds to a positive (red) or negative (blue) relationship. AM, 7:00; M, 12:00; PM, 17:00. (A) AM silage on day (d) 1. (B) M silage on d 1. (C) PM silage on d 1. (D) AM silage on d 3. (E) M silage on d 3. (F) PM silage on d 3. (G) AM silage on d 60. (H) M silage on d 60. (I) PM silage on d 60.

Microbial communities are complex multispecies assemblages that are characterized by a multitude of interspecies interactions. These bacterial interactions sustain key evolutionary and ecological processes in all environments. Recently, correlation network analysis has been applied to the silage system to detect significant patterns of copresence and mutual exclusion between bacterial taxa (14, 36). LAB is generally identified as the dominant taxa in silage fermentation as they reduce pH and inhibit the survival of undesirable microorganisms. As shown in Fig. 3, *Lactobacillus* had negative correlations with many bacterial genera in AM silage. It suggested that *Lactobacillus* had played a key role in excluding other bacteria during ensiling. This was consistent with numerous studies demonstrating that inoculation of *Lactobacillus* can easily change the bacterial community composition and their succession in silages (36, 37). In comparison, *Weissella* and *Pediococcus* were the LAB genera with notable negative correlations with other bacteria in M and PM silage, respectively. *Weissella* grows only at pH > 4.5. As ensiling progressed, the decreased abundance of *Weissella* suggests a diminished influence on controlling undesirable microorganisms. Likewise, the growth of most *Pediococcus* species will cease at pH <4.5 although some species have been shown to endure low pH (39). This suggests that it may be also not as effective as *Lactobacillus* in establishing dominance during ensiling. However, it is difficult to explain why the main LAB species that drove the succession of bacterial community varied among the three silages. This was probably related with their changes in absolute quantities in fresh SSG during the day. Since there are few *lactobacilli* on plants (38), small changes in the initial population may affect the rate and extent of *lactobacilli* to dominate the microbiota during ensiling.

**FIG 4:**
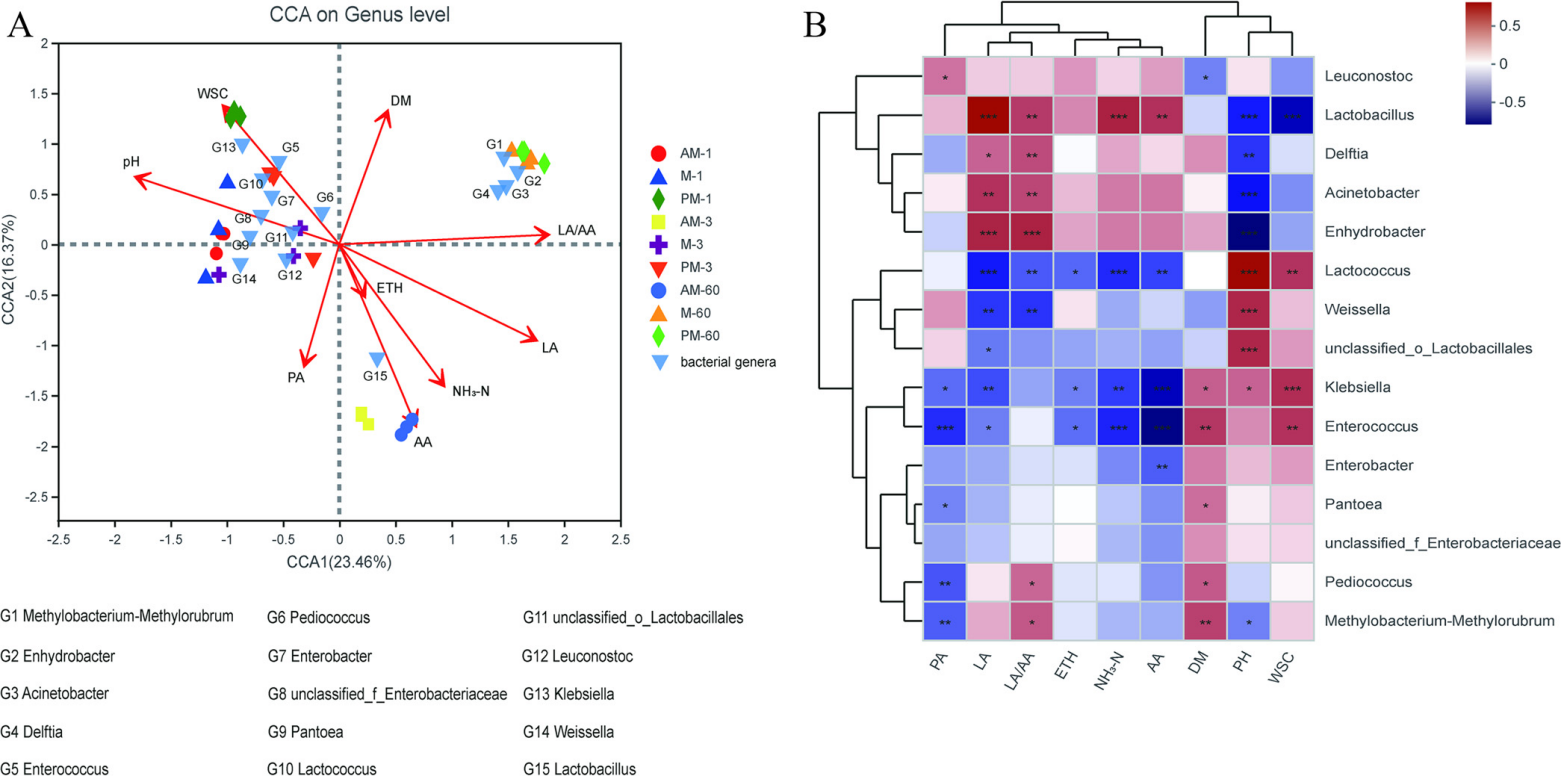
Correlation analysis of the top 15 genera with fermentation characteristics. (A) Canonical correspondence analysis (CCA) of the correlations between fermentation characteristics and bacterial community. The canonical axes are labeled with the percentage of total variance explained (%). Arrow lengths indicate the variance explained by fermentation characteristics. SSG silages harvested at various times ensiled for different days are presented as individual data points. Arabic numbers indicate days of ensiling. (B) Spearman’s correlation heatmap of the correlations between fermentation characteristics and bacterial community composition. The corresponding value of the middle heatmap is the Spearman correlation coefficient *r*, which ranges between −1 and 1, *r* < 0 indicates a negative correlation (blue), *r* > 0 indicates a positive correlation (red), and *, *P < *0.05, **, *P < *0.01, and ***, *P < *0.001, respectively. WSC, water-soluble carbohydrates; LA, lactic acid; LA/AA, lactic/acetic acid ratio; DM, dry matter; CP, crude protein; ETH, ethanol; AA, acetic acid; PA, propionic acid.

Studying the correlations between bacterial community and silage characteristics would give us a deep understanding of the key bacteria to silage quality. In the current study, canonical correspondence analysis (CCA) analysis and Spearman’s correlation heatmap were used to explore the correlations between fermentation characteristics and top 15 genera (Fig. 4A and B). The CCA suggested that there were considerable associations between silage microbial communities and fermentation parameters. The biplot score showed 23.5 and 16.4% of community differentiation by CCA1 and CCA2, respectively. The top 15 genera were divided into three clusters, and their succession was significantly related to pH (*r*^2^ = 0.708, *P = *0.001), DM (*r*^2^ = 0.337, *P = *0.011), NH_3_-N (*r*^2^ = 0.404, *P = *0.002), WSC (*r*^2^ = 0.396, *P = *0.002), lactic acid (*r*^2^ = 0.762, *P = *0.001), acetic acid (*r*^2^ = 0.814, *P = *0.001), lactic/acetic acid ratio (*r*^2^ = 0.739, *P = *0.001), and propionic acid (*r*^2^ = 0.266, *P = *0.023). The first cluster included *Lactobacillus*, and the second cluster included *Methylobacterium-Methylorubrum*, Acinetobacter, *Enhydrobacter*, and *Deftia*. The remaining genera all belonged to the third cluster. The Spearman’s correlation heatmap revealed that *Lactobacillus* was associated with high contents of acetic acid and NH_3_-N. This could be associated with the shift of *Lactobacillus* from Lactobacillus plantarum to Lactobacillus crustorum on species level during ensiling. Heterofermentative *lactobacillus* species can produce acetic acid as its main fermentation product and overcome the inhibitory effect of low pH by producing ammonia via the arginine deiminase pathway(55). Besides *Lactobacillus*, *Delftia*, Acinetobacter, and *Enhydrobacter* were also negatively correlated with pH and positively correlated with lactic acid and lactic/acetic acid ratio. Genomic analysis showed that *Delftia* has the potential to conduct l-lactic acid fermentation process using l-fucose as a carbon source (56). The positive correlation of lactic acid with *Delftia* suggested it may also have contributed to a small amount of lactic acid production at the late stage of ensiling. However, there were no reports of Acinetobacter and *Enhydrobacter* in lactic acid production. Acinetobacter and *Enhydrobacter* can tolerate extremely low pH. Their positive correlations with lactic acid might be a result of multicollinearity between pH and lactic acid variables. The cocci LAB and most enterobacteria are sensitive to pH declines (24). This explained why *Lactococcus*, *Weissella*, Enterobacter, and Klebsiella were positively correlated with pH and negatively correlated with lactic acid. *Pediococcus* strains are more tolerant of high DM conditions than other LAB (38). Positive correlation between *Pediococcus* and DM content suggests that the increased DM content of SSG in the day may have promoted the development of *Pediococcus* during ensiling.

### Kyoto Encyclopedia of Genes and Genomes metabolic pathways of bacterial community in the raw materials and silages.

**FIG 5:**
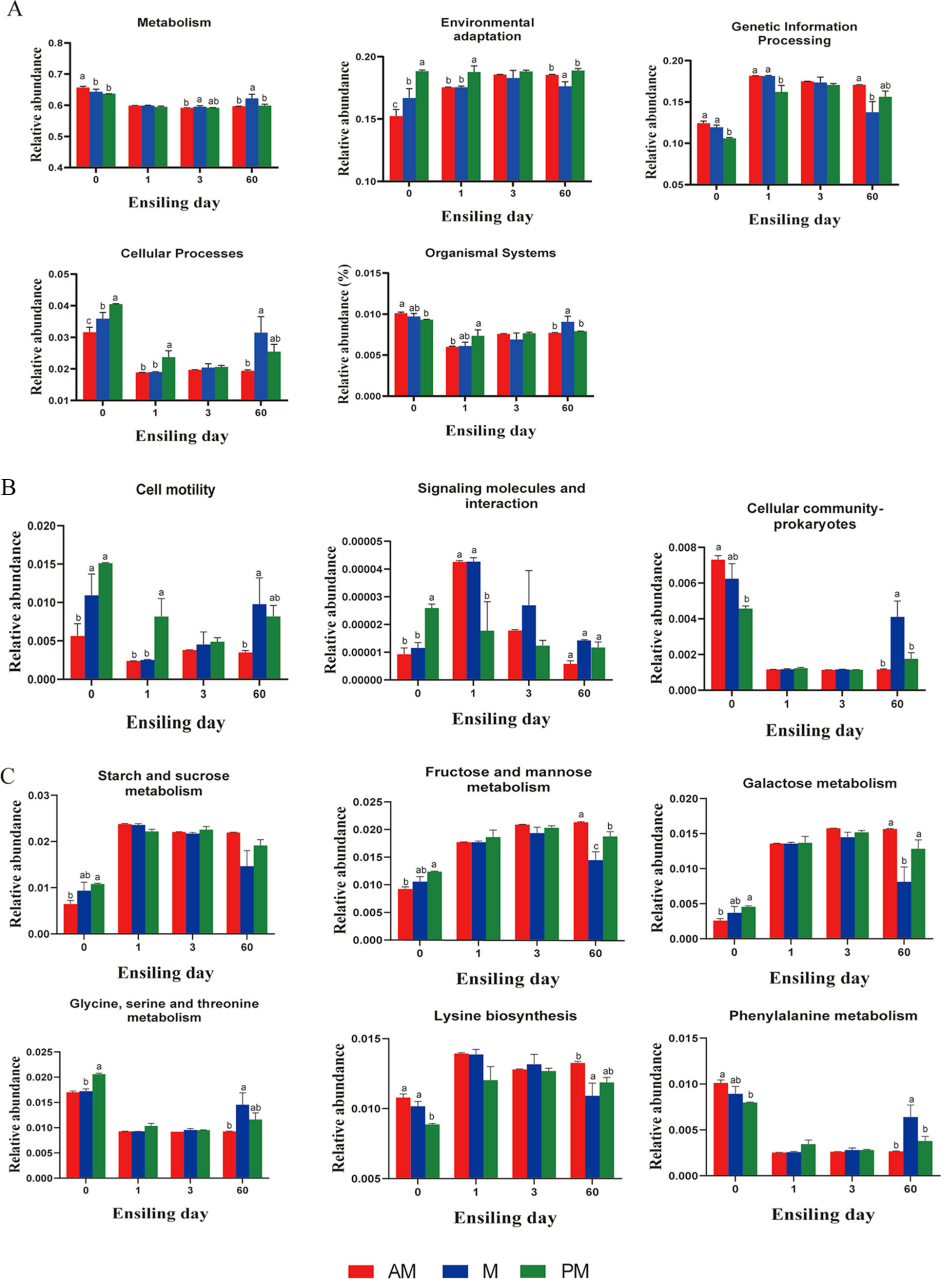
The changes of KEGG metabolic pathways on the first (A), second (B), and carbohydrate and amino acid pathways (C) on the third level obtained with Tax4Fun in raw material and silage. AM, 7:00; M, 12:00; PM, 17:00. Arabic numbers indicate days of ensiling. Error bars denote the standard error of means. Different lowercase letters (a–c) above each bar denote significant differences among harvest times according to Tukey’s test (*P < *0.05).

The fermentation process in silage is mediated by microbial activities through complicated metabolic pathways to degrade substrates or transform metabolites. Predicting the metabolic potentials is conducive to assess the influence of microbes on silage fermentation that is unable to be reflected by the dynamics of bacterial communities. Here, we used Tax4Fun tool to predict the changes in metabolic profiles of the bacterial community in raw materials and silages. The changes of Kyoto Encyclopedia of Genes and Genomes (KEGG) metabolic pathways on the first level in raw material and silage are displayed in Fig. 5A. In fresh SSG, “Metabolism,” “Genetic Information Processing,” and “Organismal Systems” of bacterial community downregulated with the delay of harvest time. The aerial part of plant is a dynamic niche where the microorganisms are exposed to fluxes in temperature, moisture, and UV during the day. The downregulation of these metabolism categories indicated that the diurnal environmental fluctuations affected the microbial survivability on leaf surface and caused a decreased level in metabolic capacity of the epiphytic microbiota. Some bacteria have adaptive strategies to deal with multiple environmental stresses. The enrichment of these bacteria might be responsible for the upregulated pathways including “Environmental adaptation” and “Cellular Processes” during the day. Ensiling downregulated “Metabolism”, “Cellular Processes”, and “Organismal Systems”, and upregulated “Genetic Information Processing”. This could be associated with the inhibition of undesirable microorganism in fresh SSG and the promotion of LAB proliferation during ensiling. When silage is well fermented and fermentation enters a stable phase, there will be minimal changes in metabolic activity in silage. At the late stage of ensiling, metabolic pathways such as “Metabolism”, “Cellular Processes”, and “Organismal Systems” upregulated in M and PM silages and the relative abundances of these pathways were highest in M silage at the late stage of ensiling. This suggested that the metabolic activities in M and PM silages was not stabilized after 60 days of ensiling.

Cell motility allows bacteria to respond to favorable or unfavorable stimuli in their environment, thereby increasing the probability of survival. In addition, the survival of all organisms depends on implementation of appropriate phenotypic responses upon perception of relevant environmental stimuli. As shown in Fig. 5B, the upregulated “Cell motility” and “Signaling molecules and interaction” on the second pathway level was consistent with the enrichment of stress-resistant bacteria in leaf surface during the day. The bacteria enriched during the day are mostly aerobes and supposed difficult to survive in silage. After 60 days of ensiling, the upregulated pathways “Cell motility”, “Signaling molecules and interaction”, and “Cellular community-prokaryotes” in M and PM silages suggested that the survival of these bacteria might be related to the enhancements in nutrient availability and cooperation among bacterial cells. It is worth noting that these metabolism categories showed highest abundances in M silage rather than in PM silage. This was probably because the harshest environment at midday have enriched the most stubborn microbiota, which are present greatest tolerance to extreme environmental stresses.

Among the KEGG metabolic pathways, carbohydrate and amino acid metabolism categories are the main metabolism pathways during silage fermentation (14). Therefore, the carbohydrate and amino acid metabolic pathways were specifically analyzed on the third pathway level (Fig. 5C). The variations of metabolic pathways among the silages on the third level was found greatest on day 1 and 60. This indicated that carbohydrate and amino acid metabolic pathways of the bacterial community varied mostly at the beginning and the end of ensiling. Ensiling enhanced the carbohydrate metabolic pathways, whereas depressed most amino acid metabolic pathways. This might be because ensiling facilitated the carbohydrate metabolism by LAB and restricted the amino acid metabolism by some undesirable microorganisms. The delayed harvest time upregulated some carbohydrate catabolic pathways in fresh SSG. This might be associated with the extensive utilization of carbohydrates by phyllosphere-adapted bacteria for producing extracellular polysaccharides (EPSs). The production of EPSs has been reported to play an important role in bacterial adaptation to different stress conditions such as desiccation (31). After 60 days of ensiling, M silage exhibited highest relative abundances of amino acid metabolisms including “Glycine, serine and threonine metabolism,” “Phenylalanine metabolism,” and lowest relative abundances of “Lysine biosynthesis.” This might be attributed to the presence of bacteria such as Acinetobacter and *Enhydrobacter*, which are able to use amino acids as carbon source (57). The threonine, phenylalanine, and lysine are essential amino acids, which animals cannot synthesize (58). The upregulated metabolism and downregulated biosynthesis of these essential amino acids in silage suggested the reductions in nutritional value for animals. Furthermore, decarboxylation of the amino acids may increase the accumulation of biogenic amines, which has adverse impacts on DM intake and palatability (59).

### Conclusion.

SSG exhibited variations in chemical composition and epiphytic microbiota during the day. These variations affected the natural fermentation parameters, bacterial community and metabolic characteristics of SSG silage. Compared to AM silage, M and PM silages exhibited delayed fermentation, unnormal lower final pH and the higher relative abundances of undesirable bacteria such as *Delftia*, *Methylobacterium-Methylorubrum*, Acinetobacter, *Enhydrobacter*, and *Bacillus*. Moreover, PM silage had highest residual WSC and M silage had highest levels of glycine, serine, threonine, and phenylalanine metabolisms at the late stage of ensiling. These results suggest that the use of SSG harvested late in the day for silage making may cause problems including declines in silage quality, potential danger to human and animals, and increased risk of aerobic instability at silage opening, while better silage characteristics could be guaranteed through morning harvest. This study would help in precise determination of the harvest schedules, providing a cost-saving and safe strategy to produce value-added silage products and supporting the shifts toward sustainable, low-cost, high-efficiency, and safety agricultural production systems.

## MATERIALS AND METHODS

### Weather conditions at harvest and silage preparation.

The SSG was grown at experimental field of Nanjing Agricultural University (32°01′19″N, 118°51′08″E, 25 m above sea level), Nanjing, China, with row width of 70 cm and an intrarow spacing of 50 cm. After 12 weeks’ growth, SSG at vegetative stage was harvested at 7:00 h (AM), 12:00 h (M), and 17:00 h (PM) on three sunny days (7, 11, and 14 July in 2021). The harvest days were randomly selected during the optimal harvest period in Jiangsu, representing three biological replicates for statistical analyses. The SSG at harvest was approximately 3.0 m in height and the stubble was 10 cm above soil level. The climatic data of harvest days including temperature, relative humidity and solar radiation intensity were obtained from the website of National Centers for Environmental Information (https://www.ncdc.noaa.gov/). As displayed in Table 4. the average temperature at AM, M and PM was 27.7°C, 30.8°C, and 29.1°C, the relative humidity was 89.4%, 74.0%, and 76.0%, and solar radiation intensity was 125, 605, and 235 W/m^2^, respectively.

**TABLE 4:** The temperature, relative humidity, and solar radiation intensity of various times on the harvest days

Item/harvest time	Harvest day			Mean
	July 15	July 16	July 19	
Temp (°C)				
7:00 (AM)	29.1	26.9	27.0	27.7
12:00 (M)	33.1	29.2	30.2	30.8
17:00 (PM)	31.4	25.5	30.4	29.1
Relative humidity (%)				
7:00 (AM)	86.9	93.8	87.4	89.4
12:00 (M)	65.2	83.7	73.2	74.0
17:00 (PM)	72.6	86.1	69.2	76.0
Solar radiation (W/m^2^)				
7:00 (AM)	145	95.2	136	125
12:00 (M)	659	312	834	602
17:00 (PM)	166	152	387	235

On each harvest day, SSG harvested at the appointed times were immediately transferred to laboratory. The harvested SSG was chopped into a theoretical length of 2 cm with a fodder chopper. After thoroughly mixed, about 200 g chopped grass were randomly taken from the composite at each harvest time during the day, packed into polyethylene plastic bags (20 × 30 cm) and vacuum sealed (DZD-400; Aomitai Technology Co, Ltd, Nanjing, China). A total of 54 bags (3 harvest times × 6 storage periods× 3 harvest days) were prepared and stored at room temperature (20 to 25°C). These bags were opened after 1, 3, 7, 14, 30, and 60 days, respectively, and sampled for further analysis.

### Experimental analysis.

The raw materials and silages were sampled for chemical and microbial composition analyses. Approximately 100-g sample was oven-dried for 48 h at 60°C for DM measurement and ground to pass 1-mm screen with a laboratory pulverizer (FW100; Taisite Instrument Co., Ltd., Tianjin, China). The DM contents were corrected with the volatile losses during oven-drying using the equations of Gallo et al. (60). TN was measured with the method of the Association of Official Analytical Chemists (61) and multiply by 6.25 to obtain CP content. The WSC content was measured by colorimetry after reaction with anthrone reagent (62). The contents of NDF and ADF were determined by the procedures of Van Soest et al. (63), with heat-stable amylase and sodium sulfite being used for NDF procedure.

To determine the ensiling traits of fresh material and fermentation parameters of silage, about 35 g of sample was blended with 60 mL distilled water and macerated for 24 h at 4°C. The extract was filtered through 2 layers of cheesecloth and a filter paper (Xinhua Co, China). The filtrate was used for pH, organic acids, ethanol, and ammonia nitrogen (NH_3_-N) determinations. The pH was measured with a HANNA HI 2221 pH meter (Hanna Instruments Italia Srl, Villafranca Padovana, Italy). The NH_3_-N was determined using the phenol-hypochlorite reaction method (62). The BC was determined according to the method of Liu et al. (64). The organic acids (including lactic, acetic, propionic, and butyric acids) and ethanol were quantified using an Agilent 1260 HPLC system equipped with a refractive index detector (Carbomix H-NP5 column, 2.5 mM H_2_SO_4_, 0.5 mL/min).

For microbial population analysis, 10 g of sample was thoroughly mixed with 90 mL of sterilized saline solution on a shaker at 120 rpm for 2 h. After that, 100 μL of solution were used and serially diluted with sterilized saline solution to 10^−2^ to ~10^−5^ for culture-medium plating. The LAB were counted on de Man, Rogosa, Sharpe medium after anaerobic conditions at 37°C for 2 days. Yeasts counts were determined on potato dextrose agar incubated at 30°C for 2 days. Aerobic bacteria were counted on nutrient agar incubated at 37°C for 24 h and total coliforms were counted on violet red bile agar at 37°C for 2 days. The remaining solution was filtered into a 50-mL centrifuge tube with 4 layers of medical gauze and stored at −80°C for DNA extraction.

### Bacterial community analysis.

The frozen solution for DNA extraction were thawed at 4°C and then centrifuged at 12,000 × *g* for 30 min to obtain a pellet for subsequent DNA extraction. The DNA extraction was conducted using the FastDNA SPIN Kit and the FastPrep Instrument (MP Biomedicals, Santa Ana, CA) according to the manufacturer’s protocols. The quantity and quality of obtained DNA were determined by NanoDrop 2000 UV–vis spectrophotometer (Thermo Scientific, Wilmington, NC). The universal primers 338F and 806R were used for the PCR amplification with the target of V3-V4 region of the bacterial 16S rRNA gene. The PCR products were purified using the AxyPrep DNA Gel Extraction Kit (Axygen Biosciences, Union City, CA) and quantified using QuantiFluor-ST (Promega, Madison, WI) according to the manufacturer’s protocol. The DNA were paired-end sequenced (2 × 300 bp) on an Illumina MiSeq PE300 platform (Illumina Inc, San Diego, CA) at Majorbio Bio-Pharm Technology Co, Ltd, Shanghai, China.

Raw sequences were processed using FLASH (version 1.2.11). The QIIME quality control process (version 1.9.1) was used to discard low-quality sequences (quality scores <20). Chimeric sequences were identified and removed using UCHIME (version 1.7.0). Only sequences at least 200-bp long after quality filtering were grouped into OTUs at 97% similarity level. The alpha-diversity estimators (Shannon, ACE, Chao1, and Coverage indexes) were analyzed using QIIME. Community structures of bacteria were analyzed from phylum to species levels using the Silva database (version 138) with a confidence threshold of 70%. PCoA was constructed to visualize the variation in bacterial communities between samples using Unifrac weighted distance metric. To illuminate the interactions among microbes, correlations of microbes were analyzed by Spearman’s rank correlations among the top 20 genera and a network was created with Ctytoscape (version 3.9.0) to visualize the correlations. CCA and Spearman’s correlation heatmap were performed by the R software (version 138) to show the relationships of bacterial community and measured variables. Functional profiles of the bacterial community were predicted based on the 16S rRNA gene sequencing data by Tax4Fun (65). The sequence data has been deposited to sequence read archive of NCBI database under BioProject PRJNA818120.

### Statistical analyses.

The Statistical Packages for the Social Sciences (SPSS, version 22) was used for data analysis. Data on chemical and microbial compositions of fresh SSG were tested to one-way analysis of variance (ANOVA), while data on fermentation parameters, chemical composition, microbial counts, and relative abundance were subjected to two-way ANOVA. Tukey’s multiple comparison was used for the means separation. Significant differences were declared when *P < *0.05.
